# Supplementary material for: Planar cell polarity: the prickle gene acts independently on both the Ds/Ft and the Stan/Fz systems
Source: Development. 2018 Sep 17;145(18):dev168112. doi: 10.1242/dev.168112 (PMC6176928; doi:10.1242/dev.168112)
Supplement: Supplementary information [file develop-145-168112-s1.pdf]

Supplementary Figures

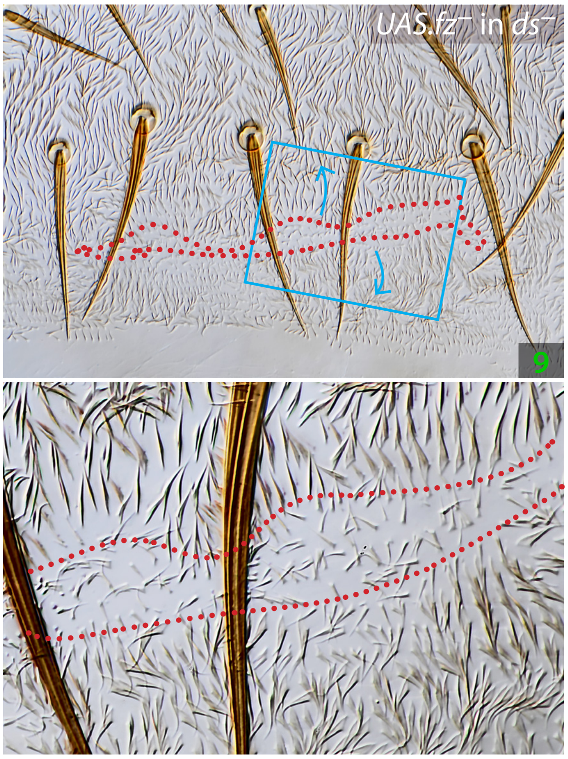

Figure S1. ***fz*-overexpressing clone in the P compartment of a *ds*⁻ fly.**
Hairs point outwards from the clone with range of 2-7 cells. Cells of the clone are marked with *pawn*, and outlined in red dots. Blue arrows indicate orientation of hairs. Blue box is enlarged below.

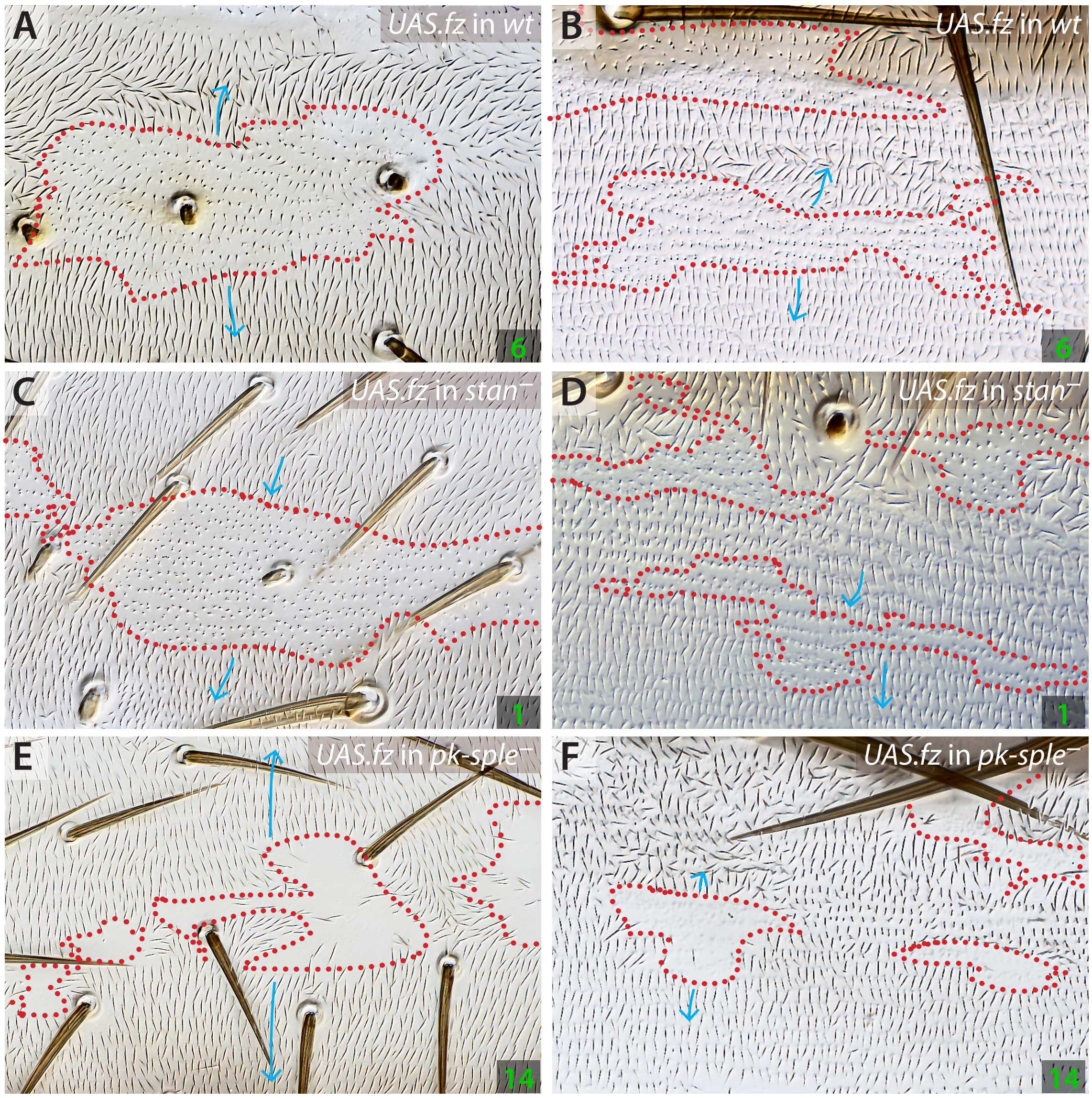

Figure S2. The effects of *fz*-overexpressing clones on various genetic backgrounds in the A and P compartments —compare with Figure 2.

A compartments (**A, C** and **E**), P compartments (**B, D** and **F**). The clones polarise responding wildtype cells outwards in both compartments (**A** and **B**). This effect is blocked when the Stan/Fz system is broken (*stan*$^-$) (**C** and **D**). In a *pk-sple*$^-$ background the sign is also outwards but the range of repolarisation is strongly reduced in the A compartment (**E** and **F**). Clones are variously marked, see Genotypes in **Materials and Methods**.

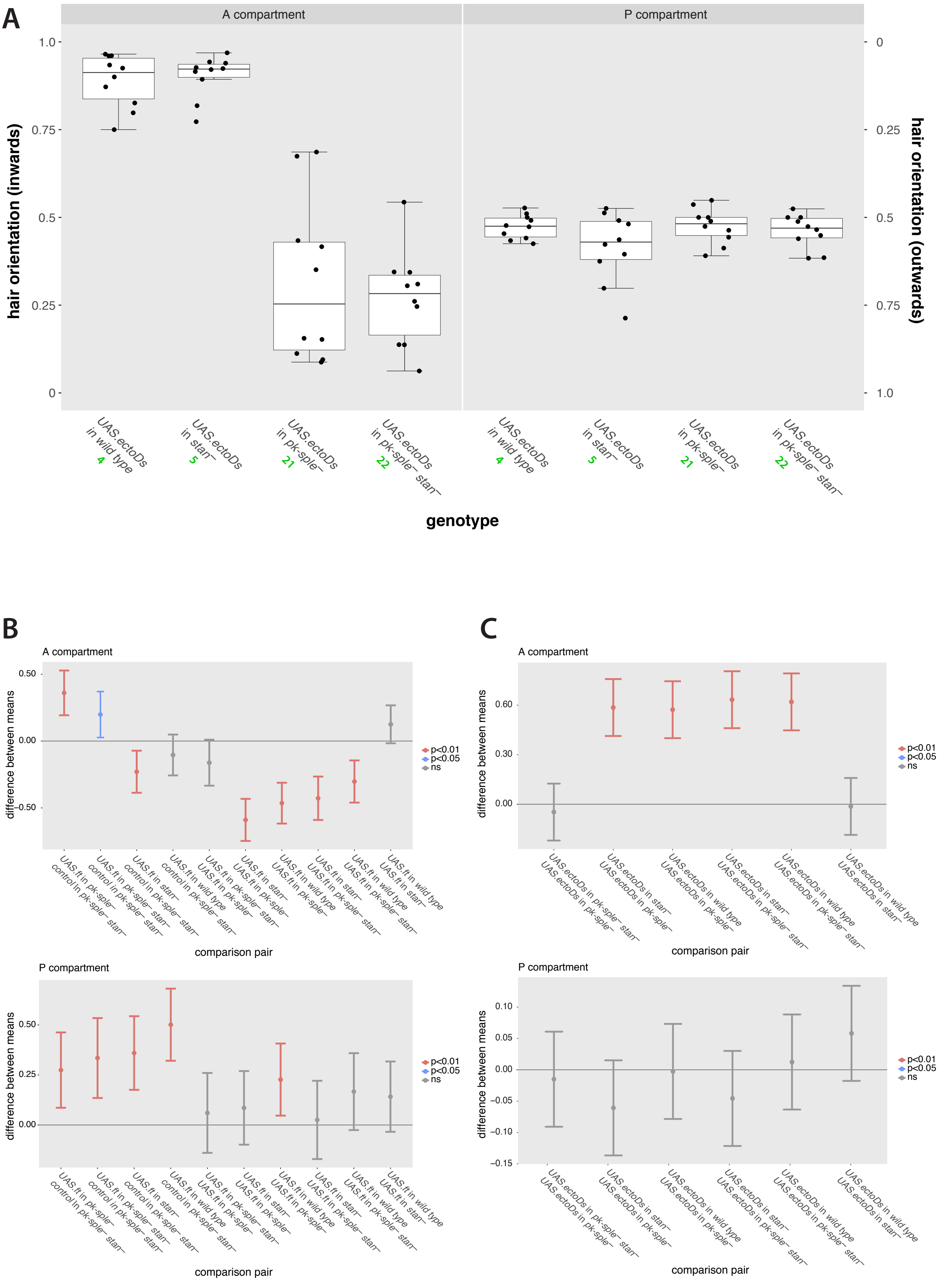

figure S3

Development • Supplementary information

Figure S3. Results of similar experiments to those in Figure 3, but here the clones were overexpressing the ectodomain of Ds.

The results are comparable with those of Figure 3 in the A compartments (although of the opposite sign to *ft*-overexpressing clones, as expected (Casal et al., 2006). None of the clones had significant effects in the P compartment — this lack of response is most simply explained by high ambient level of Ds in P, which is suggested by *ds.LacZ* expression (Casal et al., 2002). A response was visible in flies that lack *four-jointed* (*fj*) (data not shown), which increases the range of signalling by the Ds/Ft system (Casal et al., 2006). One-way Anova with post-hoc Tukey HSD analysis showing levels of significance for Figure 3 and S3, below (vertical lines are the 95% confidence intervals).

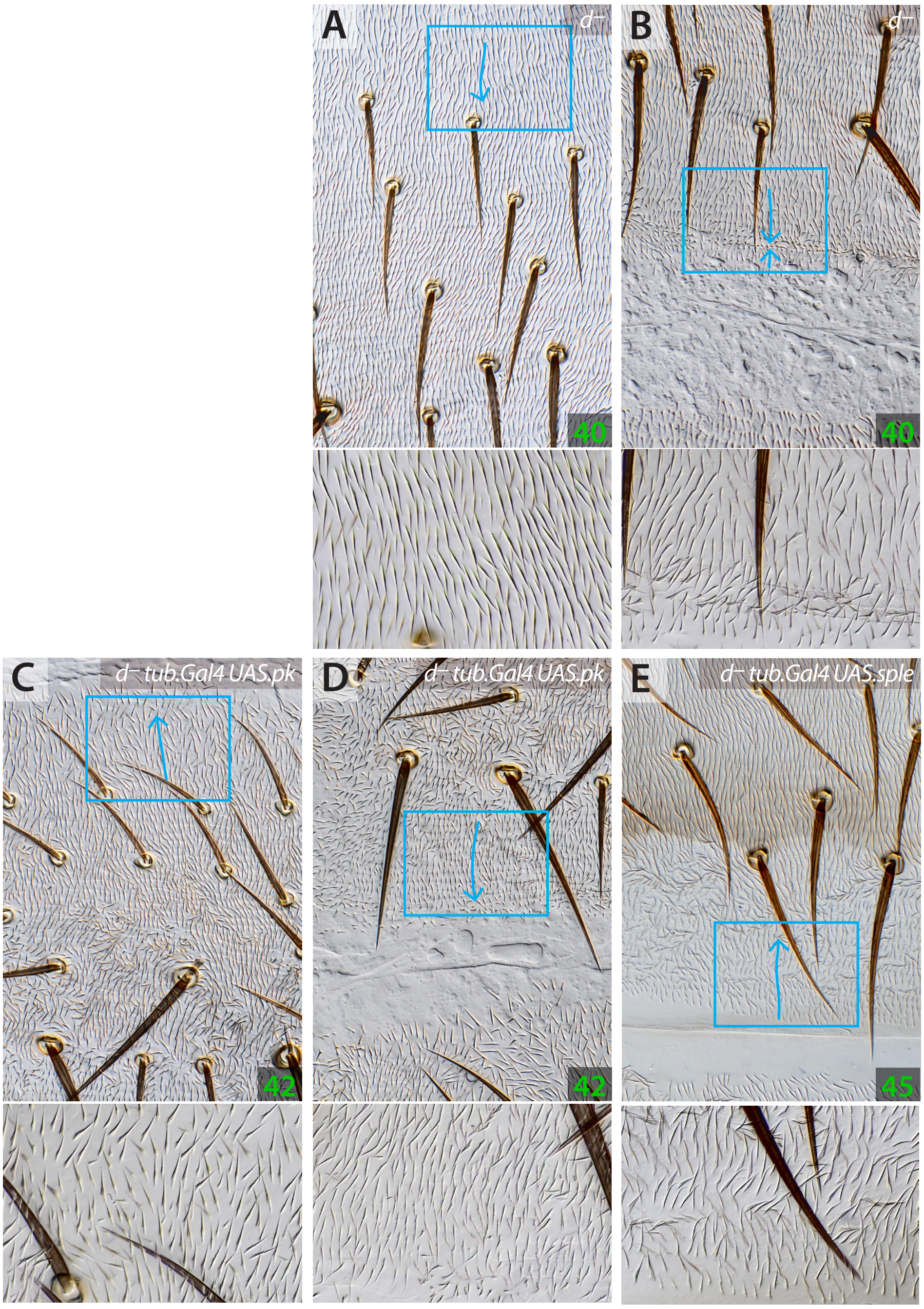

Figure S4. The effects of overexpression of *pk* and *sple* in *d*⁻ flies.
In this background the effects of extra Pk are as in *ft*⁻ *d*⁻ flies: the anterior part of the A compartment points forward and the polarity of the P compartment is "rescued" (compare **C** and **D** with **A** and **B**; see Figure 5). However extra Sple increases the area of anteriorwards polarity in the P compartment (compare **E** with **B**; see Figure 6).

Development • Supplementary information

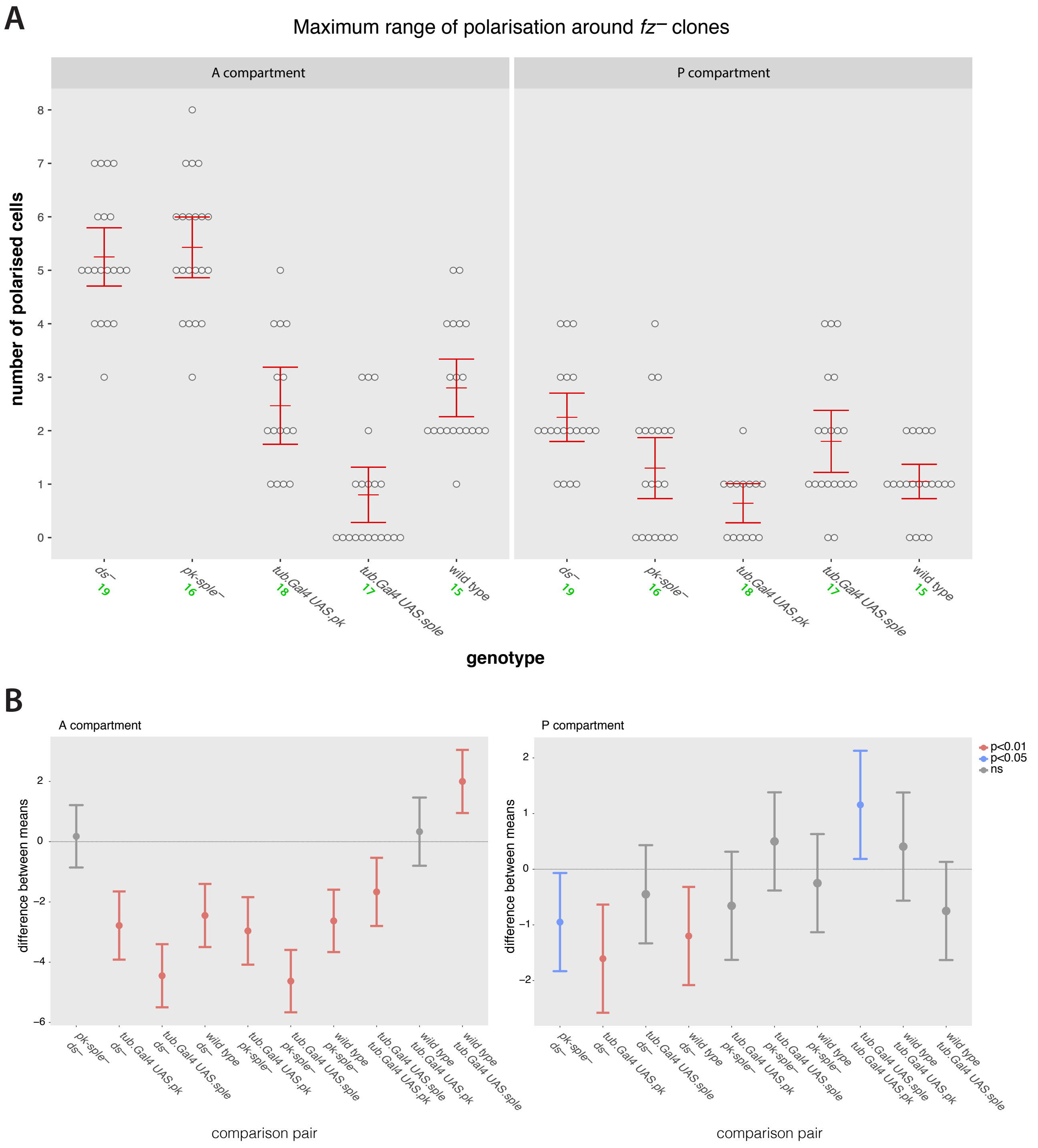

Figure S5. Range measurements for *fz*-expressing clones in wildtype and flies with a broken Ds/Ft system (*ds*⁻).

For each clonal perimeter the maximum number of cell rows showing an induced polarity change was measured. Below are the results of one-way Anova with post-hoc Tukey HSD analysis.

Development • Supplementary information

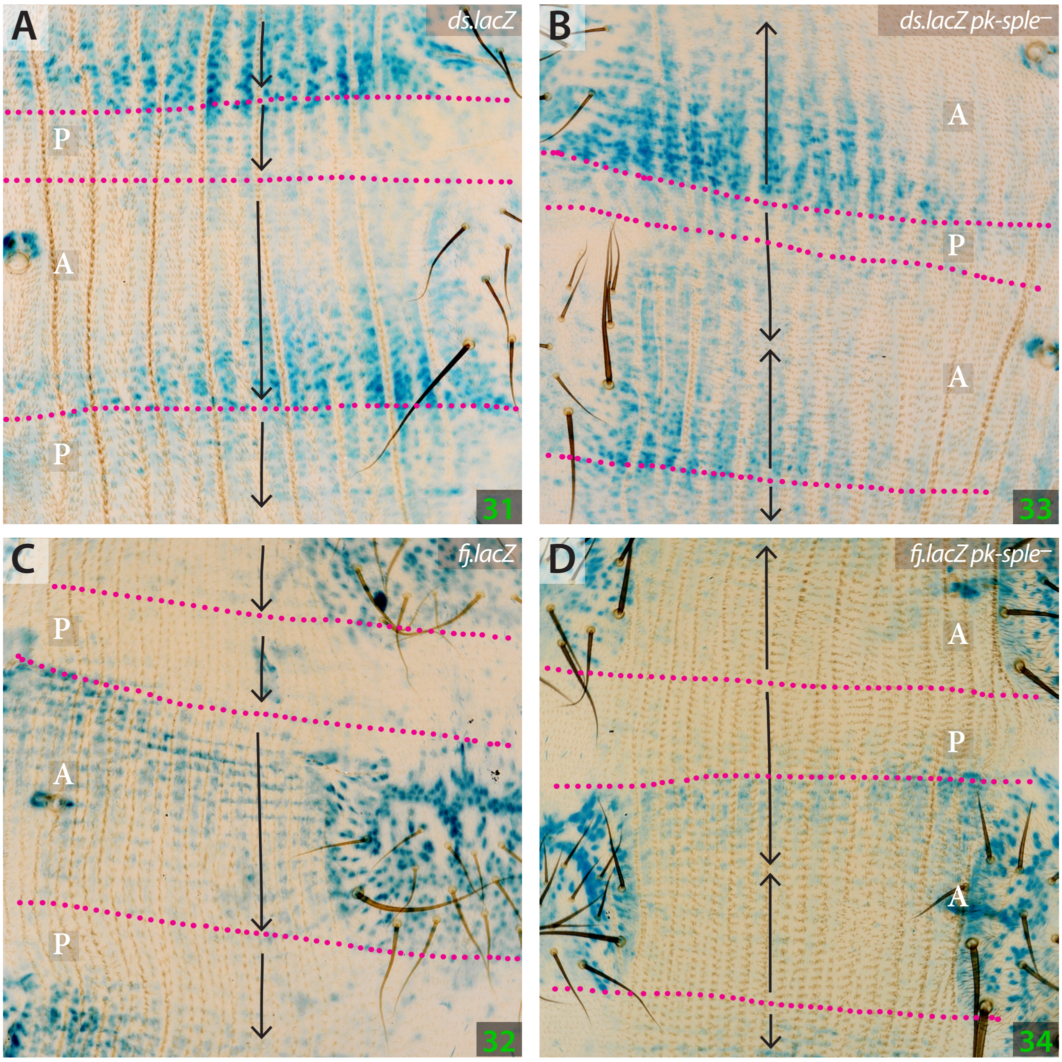

Figure S6. Ventral cuticle of the abdominal segments stained for lacZ.

A, *ds.lacZ* expression; **B**, *ds.lacZ* expression in *pk-sple*⁻; **C**, *fj.lacZ* expression; **D**, *fj.lacZ* expression in *pk-sple*⁻. Red dots delineate the approximate boundaries between the A and the P compartments. Arrows indicate the orientation of cell hairs in the pleura.

Development • Supplementary information

References

Casal, J., Lawrence, P. A. and Struhl, G. (2006). Two separate molecular systems, Dachsous/Fat and Starry night/Frizzled, act independently to confer planar cell polarity. *Development* **133**, 4561-4572.

Casal, J., Struhl, G. and Lawrence, P. A. (2002). Developmental compartments and planar polarity in *Drosophila*. *Curr. Biol.* **12**, 1189-1198.

Development • Supplementary information
